# Exosomes secreted by human-induced pluripotent stem cell-derived mesenchymal stem cells attenuate limb ischemia by promoting angiogenesis in mice

**DOI:** 10.1186/scrt546

**Published:** 2015-04-10

**Authors:** Guo-wen Hu, Qing Li, Xin Niu, Bin Hu, Juan Liu, Shu-min Zhou, Shang-chun Guo, Hai-li Lang, Chang-qing Zhang, Yang Wang, Zhi-feng Deng

**Affiliations:** Department of Neurosurgery, Shanghai Jiaotong University Affiliated Sixth People’s Hospital, 600 Yishan Road, Shanghai, 200233 China; Jiangxi Medical College of Nanchang University, 461 BaYi Avenue, Nanchang, 330006 China; Institute of Microsurgery on Extremities, Shanghai Jiaotong University Affiliated Sixth People’s Hospital, 600 Yishan Road, Shanghai, 200233 China

## Abstract

**Introduction:**

‘Patient-specific’ induced pluripotent stem cells (iPSCs) are attractive because they can generate abundant cells without the risk of immune rejection for cell therapy. Studies have shown that iPSC-derived mesenchymal stem cells (iMSCs) possess powerful proliferation, differentiation, and therapeutic effects. Recently, most studies indicate that stem cells exert their therapeutic effect mainly through a paracrine mechanism other than transdifferentiation, and exosomes have emerged as an important paracrine factor for stem cells to reprogram injured cells. The objective of this study was to evaluate whether exosomes derived from iMSCs (iMSCs-Exo) possess the ability to attenuate limb ischemia and promote angiogenesis after transplantation into limbs of mice with femoral artery excision.

**Methods:**

Human iPSCs (iPS-S-01, C1P33, and PCKDSF001C1) were used to differentiate into iMSCs in a modified one-step method. iMSCs were characterized by flow cytometry and multipotent differentiation potential analysis. Ultrafiltration combined with a purification method was used to isolate iMSCs-Exo, and transmission electron microscopy and Western blotting were used to identify iMSCs-Exo. After establishment of mouse hind-limb ischemia with excision of femoral artery and iMSCs-Exo injection, blood perfusion was monitored at days 0, 7, 14, and 21; microvessel density in ischemic muscle was also analyzed. *In vitro* migration, proliferation, and tube formation experiments were used to analyze the ability of pro-angiogenesis in iMSCs-Exo, and quantitative reverse-transcriptase polymerase chain reaction and enzyme-linked immunosorbent assay were used to identify expression levels of angiogenesis-related molecules in human umbilical vein endothelial cells (HUVECs) after being cultured with iMSCs-Exo.

**Results:**

iPSCs were efficiently induced into iMSC- with MSC-positive and -negative surface antigens and osteogenesis, adipogenesis, and chondrogenesis differentiation potential. iMSCs-Exo with a diameter of 57 ± 11 nm and expressed CD63, CD81, and CD9. Intramuscular injection of iMSCs-Exo markedly enhanced microvessel density and blood perfusion in mouse ischemic limbs, consistent with an attenuation of ischemic injury. In addition, iMSCs-Exo could activate angiogenesis-related molecule expression and promote HUVEC migration, proliferation, and tube formation.

**Conclusion:**

Implanted iMSCs-Exo was able to protect limbs from ischemic injury via the promotion of angiogenesis, which indicated that iMSCs-Exo may be a novel therapeutic approach in the treatment of ischemic diseases.

**Electronic supplementary material:**

The online version of this article (doi:10.1186/scrt546) contains supplementary material, which is available to authorized users.

## Introduction

Stem cells are undifferentiated cells that are present in the embryonic, fetal, and adult stages of life and are defined by their ability to self-renew and differentiate into multiple lineages [1, 2]. Stem cells have unique characteristics of high proliferation, specific migration, and the potential to differentiate into many different reparative or replacement cell types. Within the last few years, the important role of stem cells in the field of cell therapy has begun to be recognized, and remarkable progress in both basic research and clinical studies has confirmed that stem cells exert positive therapeutic effects in alleviating tissue injury after ischemia, including myocardial infarction [3, 4], brain ischemia [5, 6], and limb ischemia [7, 8].

It has been well established that bone marrow-derived mesenchymal stem cells (BMSCs) are an ideal cell source for autologous cell-based therapy because of their highly proliferative and self-regenerative capability, powerful plasticity, and low immunogenicity [9, 10]. However, several disadvantages restrict BMSC clinical applications in autologous transplantation: because they are adult somatic cells, the proliferation and differentiation capability of BMSCs decrease after a number of passages in culture. In addition, their proliferation and differentiation potential decline significantly with increasing age- and aging-related disorders. In addition, only a limited number of BMSCs can be obtained initially from a single donor, limiting their further application [11, 12]. Recent advances in stem cell technology have enabled the generation of patient-specific induced pluripotent stem cells (iPSCs) from adult somatic cells, and these iPSCs are able to differentiate into expandable progenitor cells and mature cells [13]. iPSCs exhibit similar properties with embryonic stem cells (ESCs) in self-renewal and differentiation capacity; one distinct advantage over ESCs is that they are patient-specific and thus theoretically can overcome the need for immunosuppression in the recipient. It has been reported that iPSCs can generate unlimited amounts of early-passage patient-specific MSCs with consistent quality. Induced pluripotent stem cell-derived mesenchymal stem cells (iMSCs) are a promising cell source for autologous cell therapies in regenerative medicine because of their more powerful therapeutic function compared with BMSCs [14, 15].

Although it has been demonstrated that MSCs exhibit advantages in cell therapy, one potential challenge is the acquisition of genetic and epigenetic alterations. After long-term culture, MSCs become immortalized and spontaneously transform on account of enhanced chromosome instability that is associated with the dysregulation of telomere activity and cell cycle-related genes, which can result in tumorigenesis when injected in multiple organs [16]. In addition, Jeong *et al*. found that the transplantation of short-term MSCs cultured into mice can form malignant tumors [17]. Thus, how to fully use the advantages of MSCs while avoiding disadvantages such as tumor formation is an important step toward applying them to clinical therapy for diseases.

Recently, accumulating evidence has indicated that stem cells exert their therapeutic action mainly via secreting molecules, such as growth factors, cytokines, chemokines, and extracellular microvesicles, into their surroundings via a paracrine mechanism [18]. Among these paracrine molecules, exosomes show unique functions in disease diagnosis and therapy [19]. Exosomes are endosomal-origin small-membrane vesicles with a diameter of 40 to 100 nm and are formed in multivesicular bodies (MVBs) by invagination of the endosomal membrane and then released into the extracellular space when MVBs fuse with the plasma membrane [20]. Recent studies have indicated that exosomes derived from BMSCs can promote angiogenesis in ischemic tissue and attenuate tissue injury after an ischemic injury [21–23]. In addition, it has been confirmed that exosomes derived from MSCs are immune-tolerant, an important property for clinical applications [24]. Thus, we hypothesized that exosomes derived from iMSCs (iMSCs-Exo) may also exhibit similar functions in reducing tissue injury after ischemia.

In the present study, we investigated the therapeutic effects of iMSCs-Exo in a mouse hind-limb ischemic model. Consistent with our hypothesis, we found that iMSCs-Exo could significantly attenuate limb ischemia. We further observed a remarkable microvessel density increase and blood perfusion recovery in the mice ischemic limb, which indicated that pro-angiogenesis may be one reason for iMSCs-Exo to alleviate ischemic diseases. An *in vitro* study demonstrated that iMSCs-Exo can promote human umbilical vein endothelial cell (HUVEC) migration, proliferation, and tube formation. Furthermore, iMSCs-Exo can promote angiogenesis-related gene expression and protein secretion in HUVECs. To the best of our knowledge, this is the first study to suggest that iMSCs-Exo also exhibit a pro-angiogenesis function, which indicates that iMSCs-Exo can become a novel regulator in iMSC-based ischemic injury therapy.

## Methods

The use of human iPSCs in this study was approved by the local ethics committee of Nanchang University (2 October 2011).

### Generation of mesenchymal stem cells from human induced pluripotent stem cells

Three human iPSC lines were used in the generation of MSCs. The first human iPSC line (iPS-S-01) was provided by the Institute of Biochemistry and Cell Biology of the Chinese Academy of Sciences in agreement with Liao and Xiao [25]. Two additional iPSC lines—iPSCs (C1P33) and iPSCs (PCKDSF001C1)—were provided by the South China Institute for Stem Cell Biology and Regenerative Medicine Group of the Chinese Academy of Sciences in agreement with Duan-qing Pei [26]. The iPSCs were routinely cultured and expanded on human ESC-Qualified BD Matrigel (BD Biosciences, Sparks, MD, USA) in six-well plates in mTESR1 (StemCell Technologies, Vancouver, BC, Canada) [27]. When iPSCs were 90% confluent, mTESR1 was replaced with MSC medium, which contained Dulbecco’s modified Eagle’s medium (DMEM)-low glucose (Corning, Tewksbury, MA, USA) supplemented with 10% fetal bovine serum (FBS) (Gibco, Grand Island, NY, USA) and 2 mM L-Glutamine [28]. The MSC medium was changed every 2 days. After 14 days in culture, the cells were trypsinized (0.25% trypsin/1 mM EDTA; Gibco) and serially reseeded thereafter in 0.1% gelatin-coated 25- and 75-cm^2^ cell culture flasks (Corning) at a density of 1 × 10^5^/mL in MSC medium. When cells were confluent in 75-cm^2^ cell culture flasks, they were considered passage 1. Usually, at passage 4, the cells demonstrated a morphology similar to that of fibroblast-like cells and were used to analyze iMSC phenotypic characteristics, differentiation potential, and further experiments.

### Flow cytometry analysis

iPSC and iMSC surface antigens were analyzed by using flow cytometry. In total, 5 × 10^5^ cells were incubated with 1% bovine serum albumin (BSA) (Gibco) for 30 minutes to block non-specific antigens. The following conjugated monoclonal antibodies (BD Biosciences) were used at the concentration recommended by the manufacturer: CD29-PE, CD34-APC, CD44-FITC, CD45-FITC, CD73-PE, CD90-PE. CD105-FITC, CD133-PE, CD146-PE, and HLA-DR-PE. Non-specific fluorescence was determined by incubation of similar cell aliquots with isotype-matched mouse monoclonal antibodies (BD Biosciences). After two washes in 1% BSA, the cells were resuspended in 200 μL of 1% BSA and analyzed by using the guava easyCyte™ system (Millipore, Billerica, MA, USA).

### Multipotent differentiation potential of induced pluripotent stem cell-derived mesenchymal stem cells

Osteogenesis, adipogenesis, and chondrogenesis were examined to determine the iMSC multipotent differentiation potential. For osteogenic induction, 5 × 10^4^ iMSCs were seeded in 24-well plates until 90% confluence and replaced with osteogenesis medium (Gibco). The cells cultured in MSC medium served as control. After 21 days, the cells were fixed with 4% paraformaldehyde and stained with Alizarin Red to detect areas of mineralized calcium. For adipogenic induction, 5 × 10^4^ iMSCs were seeded in 24-well plates until complete confluence, and differentiation was induced by adipogenesis medium (Gibco). The cells cultured in MSC medium served as the control. After 21 days, the cells were fixed with 4% paraformaldehyde and stained with Oil Red O. For chondrogenic induction, 1 × 10^6^ cells were centrifuged in a 15-mL polypropylene falcon tube to obtain a pellet, and chondrogenic medium (Gibco) was gently added to the pellet. After 21 days, the pellet was fixed with 4% paraformaldehyde and embedded in optimum cutting temperature compound (OCT) (Thermo Fisher, Waltham, MA, USA). Cryosections were stained with Toluidine Blue to detect the presence of proteoglycans. All stained cells were observed under an optical microscope (Leica, Solms, Germany).

### *In vitro* culture of human umbilical vein endothelial cells

In this study, HUVECs were isolated from human umbilical cords, which were obtained with informed consent and local ethics approval [29]. Briefly, the cords were washed twice with warmed phosphate-buffered saline (PBS) to flush out blood and clots among other factors; HUVECs were digested with 0.5 mg/mL type II collagenase (Sigma-Aldrich, St. Louis, MO, USA) for 30 minutes at 37°C and drawn out from the vessel wall by medium 200 (M200) (Gibco) containing 10% FBS; after centrifugation at 1,000 revolutions per minute for 5 minutes at room temperature, HUVECs were seeded onto 1% gelatin-coated 25-cm^2^ cell culture flasks in M200 + 10% FBS. Once HUVECs reached 90% confluence, they were further trypsinized and reseeded into 25-cm^2^ cell culture flasks and maintained in M200 supplemented with 2% low serum growth supplement (M200 + LSGS) (Cascade Biologics, Portland, OR, USA). HUVECs at passage 2 were used in the experiments as described below.

### Isolation and purification of iMSCs-Exo

Exosomes were isolated from iMSC supernatant as previously described [30, 31]. Briefly, 80% confluent iMSCs were rinsed three times with PBS and cultured for 48 hours in a chemically defined medium (MesenGro hMSC medium; stemRD, San Francisco, CA, USA). The medium was obtained and centrifuged at 300 *g* for 10 minutes and at 2,000 *g* for 10 minutes at 4°C, and the cell supernatant was filtered by using a 0.22-μm filter sterilize Steritop™ (Millipore) to remove whole cells and cellular debris. Afterwards, the supernatant was transferred to the upper compartment of an Amicon Ultra-15 Centrifugal Filter Units (Millipore) and centrifuged at 4,000 *g* at 4°C until the volume in the upper compartment was reduced to approximately 200 μL. The ultrafiltration liquid was washed twice with PBS and re-ultrafiltrated to 200 μL. For exosome purification, the liquid was laid on top of 30% sucrose/D_2_O cushion in a sterile Ultra-Clear™ tube (Beckman Coulter, Brea, CA, USA) and centrifuged at 100,000 *g* for 60 minutes at 4°C (Sorvall, Avanti J-26XP, fixed angle rotor; Beckman Coulter). The fraction containing iMSCs-Exo was recovered by using an 18-G needle and diluted in PBS and was centrifuged at 4,000 *g* at 4°C in centrifugal filter units until the final volume reached 200 μL. Exosomes were stored at −80°C or used for downstream experiments. In addition, an equal volume of serum-free medium without culturing cells was obtained in the same method of iMSCs-Exo and was centrifuged to 200 μL, which referred to the ‘control medium’. iMSCs-Exo protein content was determined by using the bicinchoninic acid assay (Thermo Fisher) as previously described [32]. BSA ranged from 2 mg/mL to 25 μg/mL to generate a calibration curve. The absorbance was read at 562 nm by using a Microplate Reader (Bio-Rad Laboratories, Berkeley, CA, USA).

### Transmission electron microscopy

Transmission electron microscopy (TEM) was used to identify the morphology of iMSCs-Exo. Briefly, iMSCs-Exo were fixed in 3% glutaraldehyde for 2 hours and washed with PBS two times. iMSCs-Exo were negatively stained with 2% uranyl acetate for 30 seconds and applied to a continuous carbon grid. iMSCs-Exo were visualized on a Hitachi H-7650 transmission electron microscope (Hitachi, Tokyo, Japan), and images were captured by using a digital camera (Olympus, Tokyo, Japan).

### Western blotting analysis

Western blotting was used to identify iMSCs-Exo markers CD63, CD81, and CD9 [33]. Briefly, 5 × protein-loading buffer was added directly to the iMSCs-Exo sample and heated at 95°C for 5 minutes. Next, iMSCs-Exo protein was loaded and resolved in 12% SDS-PAGE polyacrylamide gels. The protein sample was run at 120 V for 45 minutes and transferred onto nitrocellulose membranes (Whatman, Maidstone, Kent, UK) for 1.5 hours at 100 mA. The presence of CD63, CD81, and CD9 was assessed by exposing the membranes to primary rabbit polyclonal anti-CD63 (1:1,000), anti-CD81 (1:1,000), and anti-CD9 (1:1,000) (Abcam, Cambridge, UK). The membranes were washed three times in 1 × Tris-buffered saline with tween (TBST) for 5 minutes and incubated for 1 hour in TBST containing horseradish peroxidase-conjugated goat anti-rabbit secondary antibody (Abcam). Proteins were detected by using enhanced chemiluminescence (Thermo Fisher) and imaged by using an Image Quant LAS 4000 mini bio-molecular imager (GE Healthcare, Uppsala, Sweden).

### Mouse hind-limb ischemia model and iMSCs-Exo injection

Animal study protocols were approved by the Institutional Animal Care and Use Committee at Shanghai Jiaotong University. Unilateral hind-limb ischemia was produced by excising the common left iliac-femoral artery, from which the femoral artery merges from the proximal origin to the distal point where it bifurcates into the saphenous and popliteal arteries, and the contralateral limb was used as internal control. One day after femoral artery excision, mice (n = 20 for each group) were randomly treated with multiple intramuscular injections (four injections in the quadriceps muscle) in the ischemic leg, with the iMSCs-Exo group (200 μg of iMSCs-Exo dissolved in PBS to final volume of 200 μL) or control group (the same volume of control medium dissolved in PBS to 200 μL). To control the effect of the surgery, 20 mice received a sham operation at the same position, in which the femoral artery was exposed but not excised.

### Blood flow measurements and the functional scores

Laser-Doppler Perfusion Imaging (Moor Instruments, Devon, UK) was performed to monitor blood flow recovery in response to femoral artery excision at days 0, 7, 14, and 21 post-injection. The digital color-coded images were analyzed by comparing the blood flow ratio of the ischemic (left) to the intact (right) leg expressed as percentage perfusion. Severity of the ischemia was scored by assessment of ambulatory impairment and tissue damage as previously described [34]. Limb function of all mice was scored at the same time as blood flow measurements prior to sedation.

### Histological and immunocytochemical analysis

We performed immunohistochemistry staining and immunocytochemical staining in accordance with the protocol of the manufacturer. For immunocytochemical staining, the mice were anesthetized and sacrificed by intraperitoneally injecting an overdose of chloral hydrate at days 7, 14, and 21 post-injection. The quadriceps muscle was carefully separated and fixed with 4% paraformaldehyde overnight at 4°C and embedded in OCT. Tissue sections were pre-incubated with sodium borohydride (1 mg/mL in PBS) to reduce autofluorescence, incubated overnight in 4°C with antibodies against CD31 (1:100; Abcam) and CD34 (1:100; Abcam), and incubated for 1 hour with secondary antibody conjugated to Alexa Fluor 594 (1:200; Abcam) and Alexa Fluor 488 (1:200; Abcam), respectively. Nuclei were stained with 4, 6-diamidino-2-phenylindole (DAPI) (0.5 μg/mL; Invitrogen, Grand Island, NY, USA). Isotype control antibodies were used as negative controls. Tissues were mounted, and the images were obtained by using a fluorescence microscope (Leica). For immunohistochemistry staining, the quadriceps muscle was harvested at day 21 and fixed with paraformaldehyde and embedded in paraffin, and the sections were stained with hematoxylin and eosin and examined under a light microscope (Leica) by two pathologists who were blinded to the grouping conditions.

### Endothelial cell migration assay

The real-time cell analyzer (RTCA) migration assay and scratched wound assay were used to analyze the migration effect of iMSCs-Exo to HUVECs. The xCELLigence system (Roche Applied Sciences, Basel, Switzerland) used impedance as a readout and can continuously monitor the cellular responses of biologically active small-molecule compounds, producing time-dependent cellular response profiles. The electronic readout of cell-sensor impedance is displayed in real-time as CI, a value directly influenced by cell attachment, spreading, or cell proliferation or a combination of these. The CI value at each time point is defined as Rn-Rb/Rb, where Rn is the cell-electrode impedance of the well with the cells and Rb is the background impedance of the well with only medium [35]. HUVECs (4 × 10^4^ cells per well) were seeded into the upper chamber, and M200 containing 100 μg/mL iMSCs-Exo or control medium was added into the lower chamber. The cells were incubated at 37°C in 5% CO_2_ and monitored for 24 hours. For the scratched wound assay, 2 × 10^5^ cells were seeded into 12-well plates and maintained at 37°C to permit cell adhesion and the formation of a confluent monolayer. Next, these confluent monolayers were ‘scratch’-wounded by using the tip of a p200 pipet tip. The medium was removed and rinsed once with PBS to remove the debris and smooth the edge of the scratch and then replaced with fresh M200 + LSGS medium containing 100 μg/mL iMSCs-Exo or control medium. Wound closure was monitored by collecting digital images at 0-, 12-, and 24-hour intervals after the scratch, and digital images were captured by using an inverted microscope (Leica). The images were obtained at the same position before and after incubation. The experiment was repeated three times. The level of wound closure was assessed by the ratio of closure area to initial wound (0 hours) as follows:


where Rn represents the percentage of wound closure, An represents the residual area of wound at the metering point (nh), and A0 represents the area of initial wound (0 hours) [36].

### Endothelial cell proliferation assay

A Cell Counting Kit-8 (CCK-8) assay (Dojindo, Kyushu Island, Japan) was performed to assess cell proliferation. Briefly, HUVECs were seeded at 5 × 10^4^ cells/mL (100 μL/well) in a 96-well plate. After quiescence for 12 hours, cells were treated with M200 + LSGS containing different doses of iMSCs-Exo (0, 12.5, 25, 50, and 100 μg/mL) or control medium. At days 1, 2, 3, 4, and 5, CCK-8 solution (10 μL) was added into medium and incubated for 3 hours at 37°C. The amount of formazan dye generated by cellular dehydrogenase activity was measured for absorbance at 450 nm by using a microplate reader. The optical density values of each well represented the survival/proliferation of HUVECs. All of these experiments were performed in triplicate and repeated at least three times.

### Endothelial cell capillary-like tube formation assay

*In vitro* capillary-like tube formation was evaluated on growth factor-reduced Matrigel (BD Biosciences). At least 30 minutes before the experiment, 96-well plates were coated with Matrigel. Next, 2 × 10^4^ HUVECs were seeded onto the plated Matrigel in M200 with control medium, M200 + LSGS, M200 with 50 μg/mL iMSCs-Exo, and M200 with 100 μg/mL iMSCs-Exo. Tube formation was quantified at 4, 6, and 18 hours. At each time point, the capillary-like structures were imaged from five random microscopic fields by using an inverted microscope (Leica). Tube formations—(1) total tube length per image and (2) total branch numbers per image—were measured in a blind manner by an independent observer.

### Quantitative reverse-transcriptase polymerase chain reaction

Quantitative reverse transcriptase-polymerase chain reaction (qRT-PCR) for human-specific repeat sequences was performed as previously described [37]. To identify the expression level of Nanog, Oct4, and Msx1, total RNA of iPSCs, induced iPSCs (at days 4, 6, 8, 10, 12, and 14), and iMSCs was extracted by using Trizol reagent (Invitrogen). To test gene expression of HUVECs after treatment with iMSCs-Exo, 8 × 10^5^ HUVECs were seeded onto six-well plates and cultured with M200 + LSGS containing 50 μg/mL, 100 μg/mL iMSCs-Exo, or control medium for 24 and 48 hours, and total RNA was also extracted by using Trizol reagent (Invitrogen). The following human primers were used: Nanog, Oct4, Msx1, hypoxia-inducible factor-1α (HIF-1α), vascular endothelial growth factor-A (VEGFA), VEGFB, placental growth factor (PGF), basic fibroblast growth factor (bFGF), transforming growth factor beta 1 (TGFB1), Angiogenin (Angio), VEGF receptor 2 (kinase insert domain receptor, or KDR), bFGF receptor (bFGFR), and glyceraldehyde 3-phosphate dehydrogenase (GAPDH) (all from Sangon Biotech, Shanghai, China). The primer sequences used in this study are summarized in Table 1. Next, 1 μg of total RNA was used to generate cDNA. The cDNA was diluted 1:10 in sterilized Milli-Q water. Two microliters of diluted cDNA, 5 μL of SYBR-Green, 2.2 μL of Milli-Q water, and 0.8 μL of primer were added into a 384-well plate, and amplification was performed with a 10-minute pretreatment at 95°C, 95°C for 15 seconds, and 60°C for 1 minute (40 cycles). Each qRT-PCR was performed in triplicate for yield validation.

**Table 1:** **Primers used for quantitative reverse-transcriptase polymerase chain reaction**

Genes	Forward primer (5′-3′)	Reverse primer (5′-3′)
*h-Nanog*	TGAACCTCAGCTACAAACAG	TGGTGGTAGGAAGAGTAAAG
*h-Oct4*	CCTCACTTCACTGCACTGTA	CAGGTTTTCTTTCCCTAGCT
*h-Msx1*	CGAGAGGACCCCGTGGATGCAGAG	GGCGGCCATCTTCAGCTTCTCCAG
*h-VEGFA*	CGCTCGGTGCTGGAATTTGA	AGTGGGGAATGGCAAGCAAA
*h-VEGFB*	TGGAAGAGGAGACTGGGAGG	TAGTGAGGGGAGGAAGAGCC
*h-HIF-1α*	CCATGTGACCATGAGGAAAT	CGGCTAGTTAGGGTACACTT
*h-PGF*	AGATGAAGCCGGAAAGGTGC	TAAATACACGAGCCGGGTGC
*h-bFGF*	CAATTCCCATGTGCTGTGAC	ACCTTGACCTCTCAGCCTCA
*h-TGFB1*	TTGAGGGCTTTCGCCTTAGC	TGAACCCTGCGTTGATGTCC
*h-Angiogenin*	CTCGCTTCGGCAGCACA	GGTGGTCGGAGATTCGTAGC
*h-KDR*	GTGATCGGAAATGACACTGGAG	CATGTTGGTCACTAACAGAAGCA
*h-bFGFR*	GACGGCTCCTACCTCAA	GCTGTAGCCCATGGTGTTG
*h-GAPDH*	ATCCCATCACCATCTTCC	GAGTCCTTCCACGATACCA

### Enzyme-linked immunosorbent assay

To determine the level of angiogenic trophic factors secreted by HUVECs, 8 × 10^5^ cells were seeded onto six-well plates and cultured with M200 + LSGS containing 50 μg/mL, 100 μg/mL iMSCs-Exo, or control medium for 24 and 48 hours. At the predetermined time points, the cell supernatant was collected and centrifuged to remove cells and then stored at −80°C. VEGF, TGF-B1, and Angiogenin secreted by HUVECs were quantified by using specific VEGF, TGF-B1, and Angiogenin enzyme-linked immunosorbent assay (ELISA) kits (all from Westang Bio-tech, Shanghai, China) in accordance with the instructions of the manufacturer.

### Statistical analysis

All of the experiments were performed at least three times. The data were shown as the mean ± standard error of the mean. Unpaired Student’s *t* test was used for statistical comparison of the data. *P* values of less than 0.05 were considered statistically significant.

## Results

### Differentiation of induced pluripotent stem cells into mesenchymal stem cells

**Figure 1:**
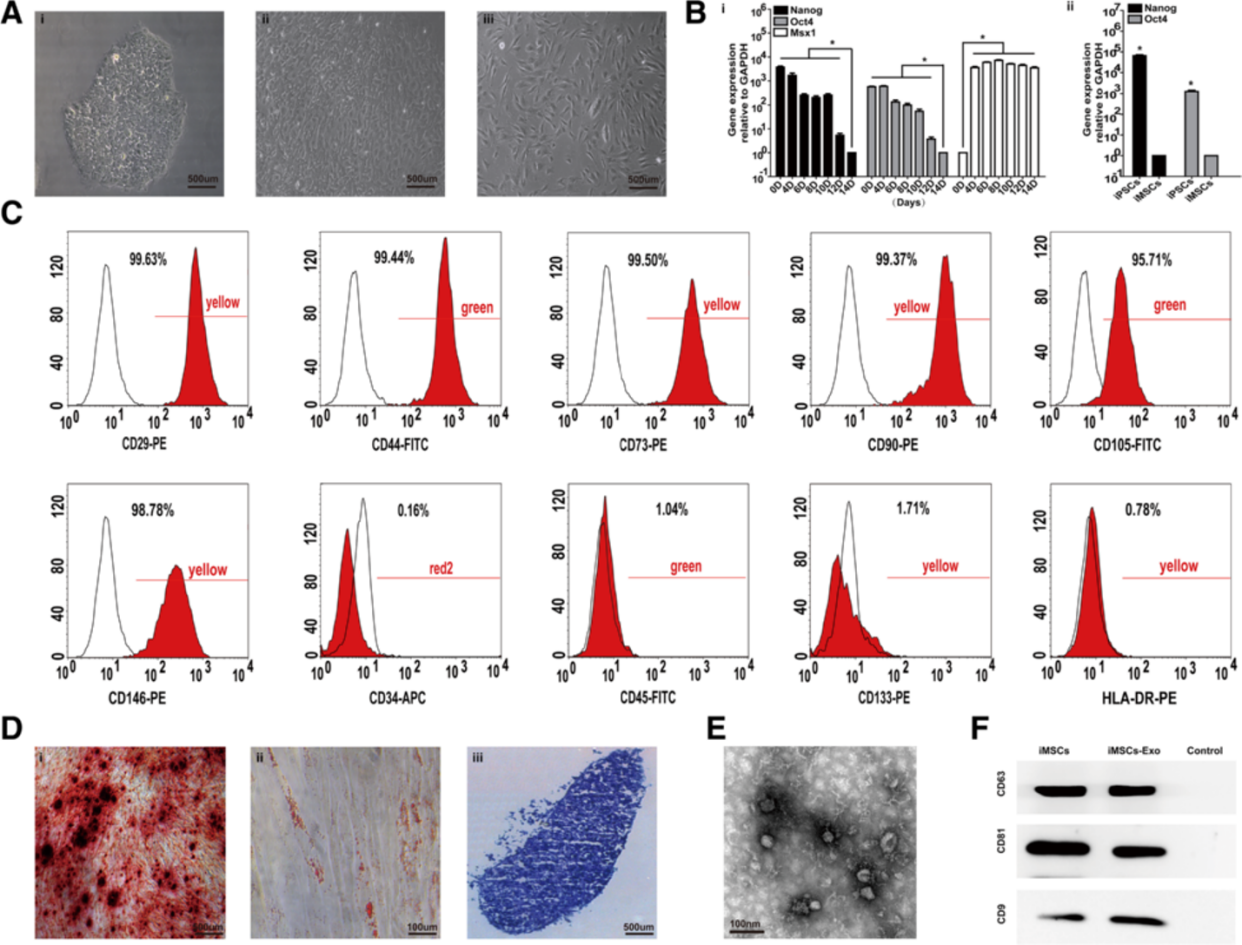
Efficient differentiation of mesenchymal stem cells (MSCs) from human induced pluripotent stem cells (iPSCs) and characterization of iMSCs-Exo. **(A)** Phase-contrast image of long-term cultured iPSC clone before differentiation (i), intermediate phase of induced iPSCs (ii), and iMSCs (iii). **(B)** Quantitative reverse-transcriptase polymerase chain reaction analysis of the expression level of pluripotent genes (Nanog, Oct4, and Msx1) in iPSCs during differentiation (i) and in iPSCs and iMSCs (ii). **(C)** Flow cytometric analysis of mesenchymal positive markers, such as CD29, CD44, CD73, CD90, CD105, and CD146, and negative markers, such as CD34, CD45, CD133, and HLA-DR. Black histograms represent the isotype controls, and the red solid peak represents the marker indicated. **(D)** Multi-differentiation potential of iMSCs. (i) Alizarin Red staining of osteogenic mineralization (day 14). (ii) Oil Red O staining of small lipid droplets (day 21). (iii) Toluidine Blue staining of cartilaginous extracellular matrix (day 21). **(E)** Morphology of iMSCs-Exo under transmission electron microscopy. **(F)** Western blotting analysis of exosomal CD63, CD81, and CD9 protein in iMSCs and iMSCs-Exo. The culture medium served as the control. GAPDH, glyceraldehyde 3-phosphate dehydrogenase; iMSC, induced pluripotent stem cell-derived mesenchymal stem cell; iMSCs-Exo, exosomes derived from induced pluripotent stem cell-derived mesenchymal stem cells.

Using a modified one-step induction protocol [28], we successfully induced iMSCs from three different iPSCs. Prior to induction, iPSCs tended to form packed clones with a high nucleus/cytoplasm ratio (Figure 1A, i). However, the iPSCs began to lose their typical morphology and formed a monolayer with a larger spindle-shaped morphology at the border of the colonies after culture in MSC medium for a few days (Figure 1A, ii). After being passaged three times, the cells exhibited a uniform fibroblastic-like morphology that resembled BMSCs (Figure 1A, iii). qRT-PCR results showed that the transcript level of pluripotency-associated genes Nanog and Oct4 were generally reduced during differentiation but that the mesoderm gene Msx1 increased rapidly to a high level (Figure 1B, i). The expression levels of Nanog and Oct4 in iMSCs were approximately 10^3–5^-fold below that in iPSCs (Figure 1B, ii).

Surface antigen profiling of iMSCs using fluorescence-activated cell sorting (FACS) analysis revealed a surface antigen profile that was qualitatively similar to that found in BMSCs, which was positive for CD29, CD44, CD73, CD90, CD105, and CD146 but negative for CD34, CD45, CD133, and HLA-DR (Figure 1C). Besides, iPSCs cultured with mTESR1 before induction were positive for CD90, weakly positive for CD29 and CD34, and negative for CD44, CD45, CD73, CD105, CD133, CD146, and HLA-DR (Additional file 1: Figure S1).The differentiation potential of iMSCs was examined by culturing in appropriate induction medium and determined by corresponding staining. Osteogenic differentiation was highly efficient: more than 90% of cells demonstrated positive staining with Alizarin Red (Figure 1D, i). After iMSCs were induced with adipogenesis induction medium for 21 days, oil droplets were observed in more than 80% of the cells (Figure 1D, ii). Chondrogenesis was also efficient: more than 90% of cells produced proteoglycans in the extracellular matrix as detected by using Toluidine Blue staining (Figure 1D, iii). Moreover, there was less than 1% positive staining in the control group. These results indicated that iMSCs were capable of multipotent differentiation.

### Characterization of iMSCs-Exo

We performed TEM to observe the morphology of iMSCs-Exo, which were isolated by using ultrafiltration combined with the purification method. TEM clearly revealed that iMSCs-Exo exhibited a cup- or round-shaped morphology with a size of 57 ± 11 nm (Figure 1E). Western blotting (Figure 1F) confirmed the expression of exosomal markers CD63, CD81, and CD9 in iMSCs-Exo.

### iMSCs-Exo promote blood perfusion and attenuate severe hind-limb ischemia

**Figure 2:**
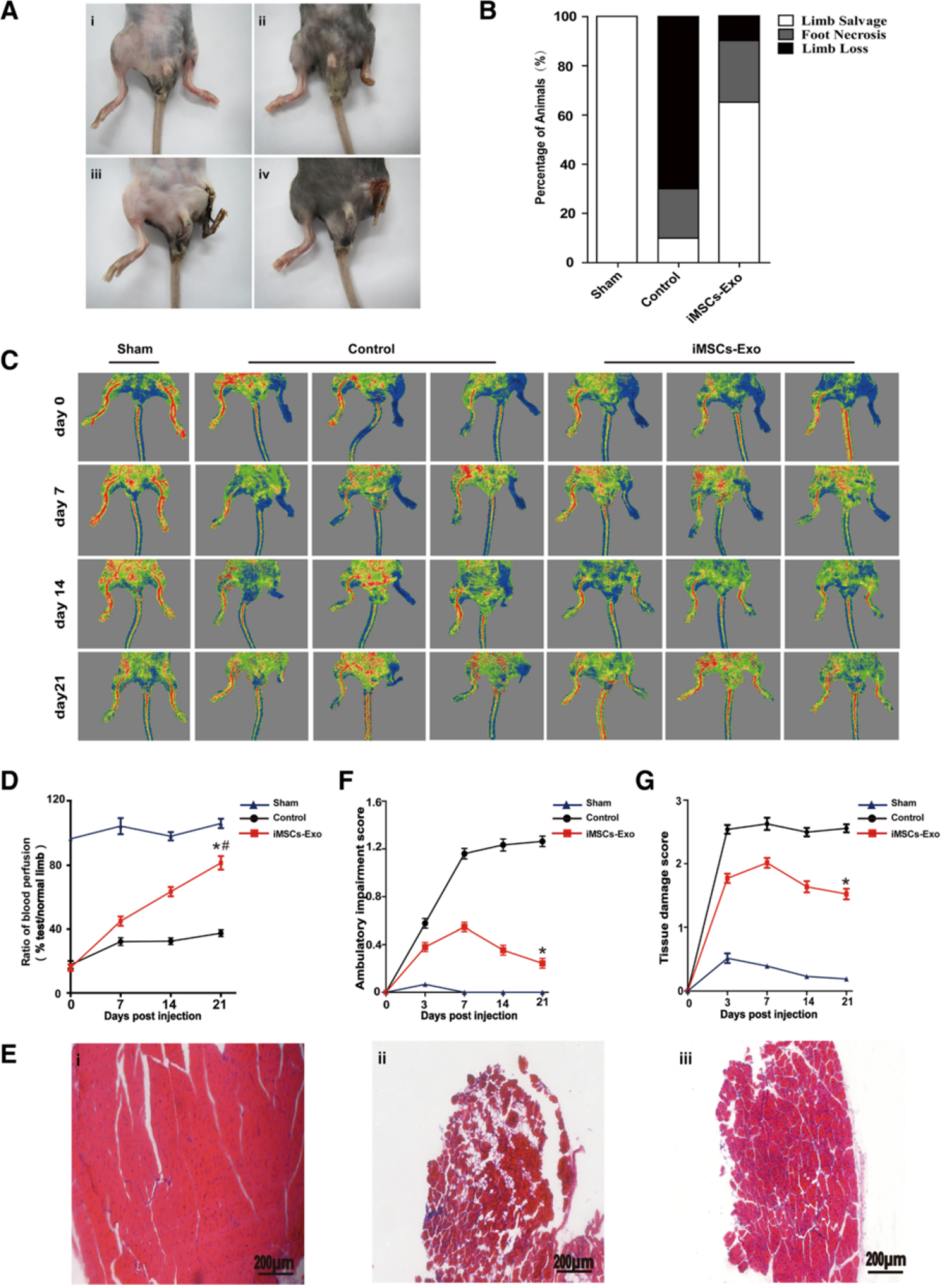
Transplantation of iMSCs-Exo improved blood flow and attenuated hind-limb ischemia. **(A)** Examples of the potential clinical outcomes after femoral artery excision: no necrosis (i), necrosis of the phalanges (ii), necrosis above the ankle joint (iii), and complete necrosis of the limb (iv). **(B)** At day 21 post-injection, the 3-group comparison showed a significant difference in the physiological status of the ischemic limb rated in three levels: limb salvage, foot necrosis, and limb loss (n = 20 per group). **(C)** Laser Doppler flow imaging showed dynamic changes in blood perfusion in limb ischemia of the 3-group at days 0, 7, 14, and 21 post-injection. **(D)** The ratio of blood flow in the left limb (ischemic) to right limb (non-ischemic) was gradually recovered in iMSCs-Exo transplanted mice at day 21 (^#^ *P* <0.05, day 0 versus 21); compared with the control group, there was significantly improvement in the ratio of blood flow at day 21 (*P <0.05, iMSCs-Exo group versus control group). **(E)** At day 21 post-injection, hematoxylin-and-eosin staining for ischemic muscle showed that muscle had structural integrity in the sham group (i), whereas in the control group, muscle had massive degeneration (ii); however, in the iMSCs-Exo group, muscle degeneration of the ischemic limb was largely protected (iii). **(F, G)** In the iMSCs-Exo group, compared with the control group, the scores of ambulatory impairment and tissue damage were significantly decreased at day 21 (*P <0.05, iMSCs-Exo group versus control group). iMSCs-Exo, exosomes derived from induced pluripotent stem cell-derived mesenchymal stem cells.

To investigate the biological functions of iMSCs-Exo, we used a mouse hind-limb ischemia model by performing an excision of the left femoral artery and injected iMSCs-Exo directly into the left quadriceps muscle. The control group was injected with control medium. After 21 days, we observed robust differences in the ischemic limb physiological status when compared with the iMSCs-Exo group, control group, and sham group (Figure 2A,B). Of the 20 mice in the control group, 14 (70%) had limb loss and four (20%) demonstrated limb necrosis, with gangrenous tissue in the thigh and calf; and limb salvage was observed in only two (10%). In contrast, of the 20 mice that received an iMSCs-Exo injection, two (10%) suffered from limb loss and five (25%) demonstrated limb necrosis in the calf and foot, and limb salvage was observed in 13 (65%). In the sham group, 20 mice recovered well with no loss or necrosis (Figure 2B).

Tissue blood perfusion and flow rate were measured by using laser Doppler at days 0, 7, 14, and 21 post-injection (Figure 2C). Repeated-measure analysis of variance (ANOVA) of blood perfusion in the 3-group comparison revealed that iMSCs-Exo promoted blood perfusion in the ischemic limb. At day 0, the iMSCs-Exo group and control group similarly demonstrated low levels of blood perfusion, and the sham group demonstrated almost the same level compared with the normal limb. At days 7 and 14, blood perfusion in the iMSCs-Exo group was gradually recovered, whereas in the control group, it was at a poor recovery level. At day 21, the difference in blood perfusion in the 3-group was prominent; blood perfusion in the iMSCs-Exo group recovered nearly the same as the normal limb, whereas blood perfusion in the control group remained at a low level. Blood perfusion in the iMSCs-Exo group was significantly improved compared with that in the control group (*P* <0.05; Figure 2D).Hematoxylin-and-eosin staining for quadriceps muscle revealed a significant difference in muscle integrity in the 3-group analyses. Muscle in the sham group showed structural integrity (Figure 2E, i). However, extensive muscle degeneration was observed in the control group when compared with the sham group (Figure 2E, ii). In the iMSCs-Exo group, in contrast with the control group, there was remarkably less muscle degeneration (Figure 2E, iii).

Limb ambulatory impairment and tissue damage score were used to evaluate ischemic limb function. Repeated-measure ANOVA analysis demonstrated that scores of ambulatory impairment and tissue damage were significantly different between the iMSCs-Exo group and control group (Figure 2F,G). At day 3, there was a similar dramatic limb function reduction in both the iMSCs-Exo group and control group, but limb function in the sham group was less injured. At day 7, limb function in the sham group recovered to a normal level, and limb function reduction in the control group was more severe compared with that in the iMSCs-Exo group (*P* <0.05). Next, we observed that limb function gradually improved in the iMSCs-Exo group after 7 days. In the iMSCs-Exo group, compared with the control group, the ambulatory impairment and tissue damage score were significantly reduced at day 21 (*P* <0.05). These data indicated that iMSCs-Exo can promote ischemic limb functional recovery.

### iMSCs-Exo promote angiogenesis in the ischemic limb

**Figure 3:**
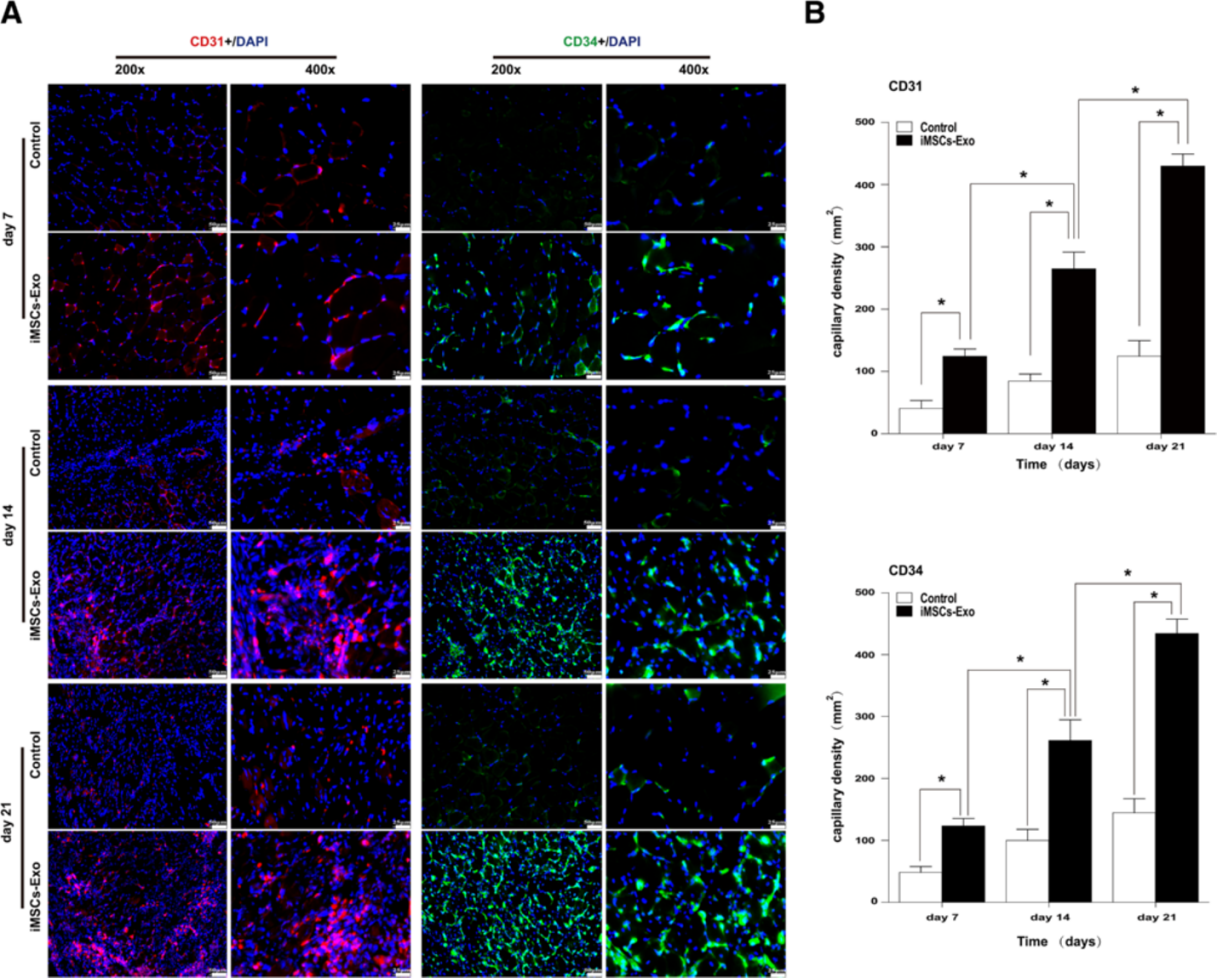
Transplantation of iMSCs-Exo stimulated angiogenesis in the ischemic limb. **(A)** Mouse endothelial cells were stained red for surface marker CD31 and green for surface marker CD34. Nuclei (blue) were stained with 4′, 6-diamidino-2-phenylindole (DAPI). Compared with the control group, the iMSCs-Exo group had a significant microvessel density improvement at days 7, 14, and 21 (3.11-, 3.14-, and 3.46-fold increase relative to the control group on days 7, 14, and 21 for CD31 staining, and 2.56-, 2.64-, and 3.01-fold increase relative to the control group on days 7, 14, and 21 for CD34 staining, respectively; *P <0.05). **(B)** Microvessel density in the control group rarely increased; however, in the iMSCs-Exo group, there was a significant increase at days 7, 14, and 21 (*P <0.05). iMSCs-Exo, exosomes derived from induced pluripotent stem cell-derived mesenchymal stem cells.

Muscle recovery after ischemia is predominantly dependent on angiogenesis because neovascularization provides an exchange of nutrients and oxygen. Thus, in this study, we used a morphometric analysis of immunohistochemical staining for the endothelial markers CD31 and CD34 to determine whether iMSCs-Exo can stimulate angiogenesis in ischemic muscle. As shown in Figure 3A and B, representative images of CD31 staining in the quadriceps muscle demonstrated that iMSCs-Exo significantly increased the average microvessel density (the number of microvessels per square millimeter of area) compared with the control group (3.11-, 3.14-, and 3.46-fold increase relative to the control group on days 7, 14, and 21, respectively; *P* <0.05). Representative images of CD34 staining also show an increase in average microvessel density compared with the control group (2.56-, 2.64-, and 3.01-fold increase relative to the control group on days 7, 14, and 21, respectively; *P* <0.05). In addition, after injection of iMSCs-Exo, the average microvessel density was increased in a time-dependent manner (*P* <0.05). These results suggested that transplantation of iMSCs-Exo can stimulate angiogenesis in the ischemic muscle.

### iMSCs-Exo promote endothelial cell migration, proliferation, and tube formation

**Figure 4:**
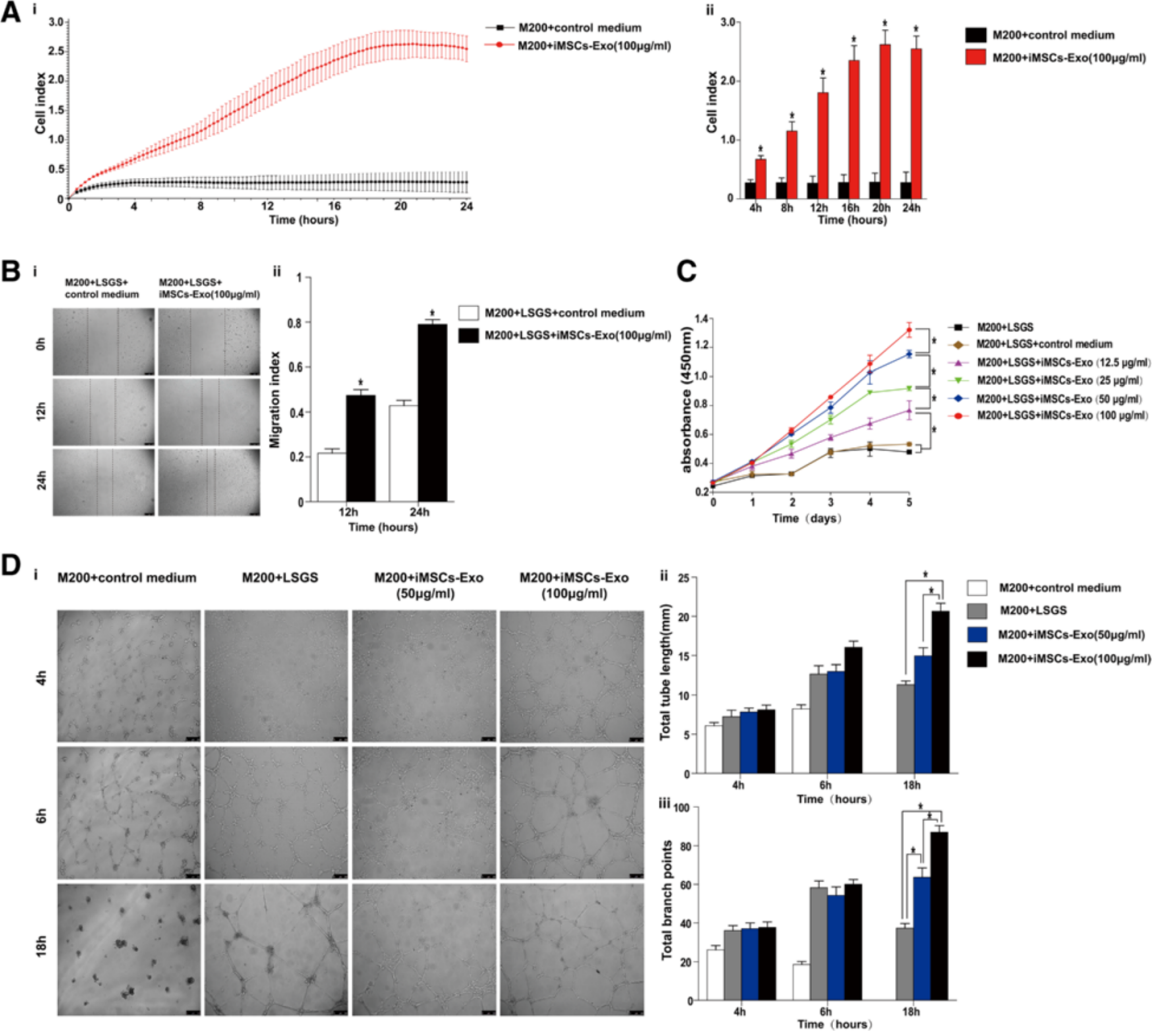
iMSCs-Exo regulated human umbilical vein endothelial cell (HUVEC) migration, proliferation, and tube formation. Migration of HUVECs was measured by using Real-Time Cell Analyzer **(A**, i, curve graph of RTCA; ii, Quantitative analysis of cell index at 4 h, 8 h, 12 h, 16 h, 20 h, 24 h for i.**)** and scratched wound assay **(B**, i, optical micrographs of scratched wound assay; ii, quantitative analysis of migration index at 12 h and 24 h for i.**)**. Compared with the control group, iMSCs-Exo could improve the migration level of HUVECs (*P <0.05). Proliferation was measured by using the Cell Counting Kit-8 (CCK-8) **(C)**. iMSCs-Exo can significantly stimulate HUVEC proliferation in a dose-dependent manner (*P <0.05). Tube formation test was performed on growth factor-reduced Matrigel **(D**, i, optical micrographs of tube formation assay; ii, quantitative analysis of total tube length at 4 h, 6 h, 18 h for i; iii, quantitative analysis of total branch points at 4 h, 6 h, 18 h for i.**)**. There was no significant difference in branch number and total tube length of the capillary-like structures for HUVECs cultured in low serum growth supplement (LSGS), 50 μg/mL, or 100 μg/mL iMSCs-Exo at 4 and 6 hours (*P* >0.05). However, after being cultured for 18 hours, HUVECs cultured in 100 μg/mL iMSCs-Exo formed more capillary-like branches (*P <0.05), and the total tube length of the capillary-like structures was much longer than that cultured in LSGS or 50 μg/mL iMSCs-Exo (*P <0.05). HUVECs cultured in control medium hardly formed capillary-like structures. iMSCs-Exo, exosomes derived from induced pluripotent stem cell-derived mesenchymal stem cells; M200, Medium 200.

We further investigated whether iMSCs-Exo can affect HUVEC migration, proliferation, and tube formation. The RTCA assay showed that iMSCs-Exo can significantly enhance the motility of HUVECs (*P* <0.05, Figure 4A, i and ii). This result was confirmed by the scratch wound assay (*P* <0.05, Figure 4B, i and ii). As shown in Figure 4C, iMSCs-Exo stimulated endothelial cell proliferation in a dose-dependent manner. HUVECs cultured with iMSCs-Exo showed a more enhanced proliferation ability compared with HUVECs cultured with LSGS or control medium (*P* <0.05). Furthermore, we employed a three-dimensional Matrigel assay to examine the potential effects of iMSCs-Exo on tube formation (Figure 4D, i to iii). Compared with the 4-group, HUVECs cultured in LSGS and iMSCs-Exo formed capillary-like structures, whereas HUVECs cultured in the control medium rarely formed tube structures. Moreover, there were no significant differences in tube length and branch numbers in the LSGS group and iMSCs-Exo group at 4 or 6 hours (*P* >0.05). However, after 18 hours of culture, capillary-like structures in the iMSCs-Exo group remained, whereas in the LSGS group, the tube structures degraded. The tube length and branch numbers in the 100 μg/mL iMSCs-Exo group were significantly higher compared with the 50 μg/mL iMSCs-Exo group or LSGS group (*P* <0.05). Because endothelial cell migration, proliferation, and tube formation are key processes in angiogenesis, these results demonstrated that iMSCs-Exo had the potential to promote angiogenesis.

### iMSCs-Exo promote the expression of angiogenesis-related molecules in human umbilical vein endothelial cells

**Figure 5:**
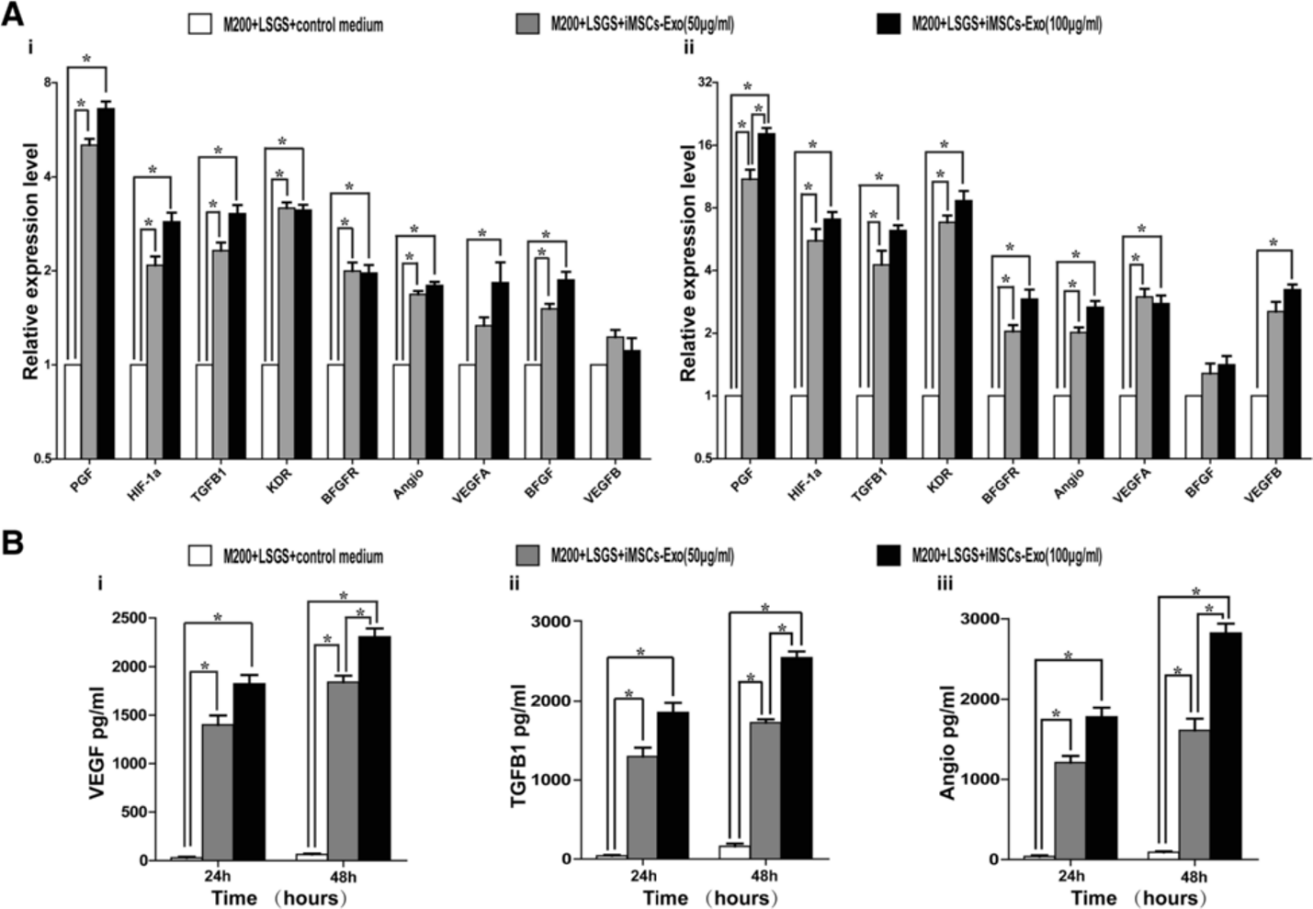
The expression level of angiogenesis-related molecules in human umbilical vein endothelial cells (HUVECs) increased after culturing with iMSCs-Exo. Quantitative reverse-transcriptase polymerase chain reaction analysis of the expression level of angiogenesis-related genes **(A)** and proteins **(B)** after culturing for 24 and 48 hours. (A) Compared with the control group, iMSCs-Exo upregulated angiogenesis-related gene expression in a dose-dependent manner (*P <0.05) at 24 hours (i) and 48 hours (ii). (B) Compared with the control group, vascular endothelial growth factor (VEGF) (i), transforming growth factor beta 1 (TGFB1) (ii), and Angiogenin (iii) secreted by HUVECs significantly increased after culture with iMSCs-Exo (*P <0.05). angio, Angiogenin; BFGF, basic fibroblast growth factor; BFGFR, basic fibroblast growth factor receptor; HIF-1α, hypoxia-inducible factor-1α; iMSCs-Exo, exosomes derived from induced pluripotent stem cell-derived mesenchymal stem cells; KDR, kinase insert domain receptor; LSGS, low serum growth supplement; M200, Medium 200; PGF, placental growth factor.

Because exosomes can deliver proteins and genetic information to recipient cells to regulate gene transcription and translation, we used qRT-PCR and ELISA to detect the expression level of angiogenesis-related genes (HIF-1α, VEGFA, VEGFB, PGF, bFGF, TGFB1, Angiogenin, KDR, and bFGFR) and proteins (VEGF, TGFB1, and Angiogenin) after HUVECs were cultured with iMSCs-Exo or control medium for 24 and 48 hours. As shown in Figure 5A (i and ii), iMSCs-Exo could promote angiogenesis-related gene expression (*P* <0.05). Moreover, iMSCs-Exo stimulated gene expression in a dose-dependent manner. As shown in Figure 5B (i to iii), HUVECs in the iMSCs-Exo group secreted more VEGF, TGFB1, and Angiogenin compared with HUVECs in the control medium group (*P* <0.05), and iMSCs-Exo stimulated VEGF, TGFB1, and Angiogenin secretion also in a dose-dependent manner. Taken together, these results demonstrated that iMSCs-Exo can promote angiogenesis-related gene expression and then protein secretion in a dose-dependent manner.

## Discussion

In the present study, we demonstrated for the first time that transplantation of iMSCs-Exo could attenuate limb injury in a mouse hind-limb ischemic model. We found that iMSCs-Exo can robustly promote angiogenesis in ischemic muscle and improve blood perfusion in the ischemic limb, which were necessary for limb functional recovery. Furthermore, we demonstrated that iMSCs-Exo can stimulate HUVEC migration, proliferation, and tube formation. Moreover, iMSCs-Exo can activate the expression of angiogenesis-related molecules in HUVECs. Taken together, these results suggested that iMSCs-Exo can attenuate hind-limb ischemic injury by promoting angiogenesis.

With the development of regenerative medicine, stem cell transplantation is considered to have great potential in treating ischemic diseases. Many studies have demonstrated that transplanting isolated primary stem cells have positive therapeutic effects in clinical studies and experimental ischemic models [38, 39]. In the stem cell therapeutic field, adult MSCs have been largely studied with regard to their high self-renewal capacity, differentiation capacity, and low immunogenicity. Among all multipotent MSCs, those derived from bone marrow have recently emerged as an attractive cell type for the treatment of ischemic diseases [40, 41]. However, large-scale application of autologous BMSCs still presents challenges because patients can offer only a limited number of BMSCs. In addition, BMSC proliferation and differentiation potential significantly decline with the increasing age- and aging-related disorders [11, 12]. Over the past few years, remarkable progress has been made in the generation of clinically compliant and safer human iPSCs by using virus- or vector-free methods or both [42, 43]. iPSCs become a promising cell source for clinical application because they can be generated from the patients’ somatic cells, which avoid the ethical issues and immune rejection. Moreover, previous study has shown that MSCs derived from iPSCs exhibited greater proliferative capacity than primary cultures of BMSCs [15]. In the present study, we adopted a modified one-step method to induce iPSCs into iMSCs, which displayed typical mesenchymal characteristics. In contrast to iPSCs and ESCs, iMSCs expressed no pluripotency genes, such as Nanog and Oct4, which are thought to be essential for maintaining the function of iPSCs and ESCs [44].

Currently, an increasing number of studies have reported that improvement of tissue damage by stem cells cannot be attributed to the differentiation of the delivered stem cells to replace injured cells. Their results indicated that the beneficial effects of stem cell therapies occur via secretory molecules in addition to cell replacement, a process known as the paracrine effect [18]. Stem cells release growth factors, cytokines, chemokines, and extracellular microvesicles into their surroundings, which subsequently benefit cell regeneration or angiogenesis [45]. Exosomes secreted by stem cells become an important active component in the ‘paracrine hypothesis’ for their unique characteristics and positive effects in the treatment of ischemic injury [19, 46].

Many studies have shown that exosomes derived from BMSCs can alleviate tissue damage and promote functional recovery after ischemic injury [21, 23]. Because iMSCs have been demonstrated to have a strong therapeutic function, we proposed that iMSCs-Exo may also have a powerful function in attenuating injury after ischemia. We performed *in vivo* and *in vitro* experiments to prove our hypothesis. Firstly, we used ultrafiltration combined with a purification method to isolate iMSCs-Exo. These iMSCs-Exo with a diameter of no more than 100 nm (57 ± 11 nm) expressed the exosome-specific surface markers CD63, CD81, and CD9. Secondly, we administered an intramuscular injection of iMSCs-Exo into mice with ischemic limb to examine their therapeutic function. Consistent with our hypothesis, mice in the iMSCs-Exo-treated group showed more limb salvage and a slightly lesser degree of necrosis compared with the control group. In addition, the ischemic limb treated with iMSCs-Exo presented more abundant blood perfusion, with higher microvessel density in ischemic muscle, which indicated that iMSCs-Exo can promote angiogenesis to attenuate limb injury. Thirdly, iMSCs-Exo promoted HUVEC migration, proliferation, and tube formation as demonstrated *in vitro* angiogenesis experiments, which further confirmed the function of iMSCs-Exo in promoting angiogenesis.

Exosomes are an integral part of the intercellular microenvironment and act as regulators of cell-to-cell communication. They can stimulate specific receptors in recipient cells directly or deliver proteins and genetic information into recipient cells after being internalized. These proteins or miRNAs can alter the bioactivity of recipient cells via the activation of different signaling pathways and regulation of protein translation [47]. Many reports have shown that exosomes derived from MSCs contain various proteins and miRNAs. Proteomic analysis showed that exosomes derived from MSCs harbor growth factors, cytokines, signaling molecules, and adhesion molecules, such as VEGF, TGFB1, and interleukin-8 (IL-8), which have been proven to contribute to the pro-angiogenic activity [48]. miRNA microarray results also showed that exosomes derived from MSCs contain many miRNAs, including miR210, miR126, miR132, and miR21, which have been proven to play important roles in angiogenesis [49]. Umezu *et al*. demonstrated that exosomes derived from multiple myeloma cells can transfer miR135b to endothelial cells to directly suppress factor-inhibiting hypoxia-inducible factor 1 (FIH-1), and activate HIF-1α via the HIF-FIH signaling pathway, leading to the overproduction of angiogenic cytokines such as VEGF, angiopoietin-1, and osteopontin, therefore resulted in endothelial cell migration, proliferation, and angiogenesis [50]. Tadokoro *et al*. reported that exosomes derived from hypoxic K562 cells transfer miR210 into endothelial cells to downregulate Ephrin-A3 (EFNA3) gene expression and thereby enhance tube formation of endothelial cells [51]. A recent study by Sheldon *et al*. has shown that endothelial exosomes transfer Delta-like ligand 4 (Dll4) protein to neighboring endothelial cells, leading to an inhibition of Notch signaling and an increased capillary-like structure formation *in vitro* and *in vivo*[52]. Therefore, these results indicate that angiogenesis-related proteins and miRNAs may be the main components in exosomes to exert their pro-angiogenesis function.

In our experiment, we found that iMSCs-Exo can stimulate angiogenesis-related gene expression and protein secretion. After being cultured with iMSCs-Exo, HUVECs expressed higher levels of PGF, HIF-1α, TGFB1, VEGFA, VEGFB, Angiogenin, bFGF, KDR, and bFGFR and secreted more VEGF, TGFB1, and Angiogenin. These data indicated that iMSCs-Exo can activate an array of angiogenesis-related gene expression and protein secretion after their uptake by HUVECs. It has been well demonstrated that simultaneous delivery of multiple angiogenic factors is more effective than the delivery of a single angiogenic factor in enhancing vessel density and maturity [53], which suggests that iMSCs-Exo may play a more powerful pro-angiogenesis function than that of a single recombinant angiogenic factor supplement.

## Conclusions

The present study demonstrated that iMSCs-Exo transplantation can protect against ischemic injury in an experimental mouse hind-limb ischemic model. One potential mechanism for exerting the protective function of iMSCs-Exo was the stimulation of angiogenesis in ischemic muscle. Overall, the application of iMSCs-Exo may be a novel therapeutic approach in the treatment of ischemic disease.

## Electronic supplementary material

Additional file 1: Figure S1: Flow cytometric analysis of mesenchymal markers of induced pluripotent stem cells (iPSCs). Flow cytometric analysis revealed that iPSCs were positive for CD90, weakly positive for CD29 and CD34, and negative for CD44, CD45, CD73, CD105, CD133, CD146, and HLA-DR. Black histograms represent the isotype controls, and the red solid peak represents the indicated marker. (DOCX 617 KB)

## Abbreviations

ANOVA: analysis of variance
APC: allophycocyanin
bFGF: basic fibroblast growth factor
bFGFR: basic fibroblast growth factor receptor
BMSC: bone marrow-derived mesenchymal stem cell
BSA: bovine serum albumin
CCK-8: Cell Counting Kit-8
CI: cell-sensor impedance
ELISA: enzyme-linked immunosorbent assay
ESC: embryonic stem cell
FBS: fetal bovine serum
FIH: factor-inhibiting hypoxia-inducible factor
FITC: fluorescein isothiocyanate
HIF-1α: hypoxia-inducible factor-1α
HUVEC: human umbilical vein endothelial cell
iMSC: induced pluripotent stem cell-derived mesenchymal stem cell
iMSCs-Exo: exosomes derived from induced pluripotent stem cell-derived mesenchymal stem cells
iPSC: induced pluripotent stem cell
KDR: kinase insert domain receptor
LSGS: low serum growth supplement
M200: Medium 200
MSC: mesenchymal stem cell
MVB: multivesicular body
OCT: optimum cutting temperature
PBS: phosphate-buffered saline
PE: phycoerythrin
PGF: placental growth factor
qRT-PCR: quantitative reverse-transcriptase polymerase chain reaction
RTCA: real-time cell analyzer
TBST: Tris-buffered saline with tween
TEM: transmission electron microscopy
TGFB1: transforming growth factor beta 1
VEGF: vascular endothelial growth factor.
