# Supplementary material for: Exosomes secreted by human-induced pluripotent stem cell-derived mesenchymal stem cells attenuate limb ischemia by promoting angiogenesis in mice
Source: Stem Cell Res Ther. 2015 Apr 10;6(1):10. doi: 10.1186/scrt546 (PMC4533800; doi:10.1186/scrt546)

**Additional file 1**

**Figure S1. Flow cytometric analysis of mesenchymal markers of iPSCs.** Flow cytometric analysis revealed iPSCs were positive for CD90, weakly positive for CD29, CD34, while negative for CD44, CD45, CD73, CD105, CD133, CD146, and HLA-DR. Black histograms represent the isotype controls and the red solid peak represents the indicated marker.


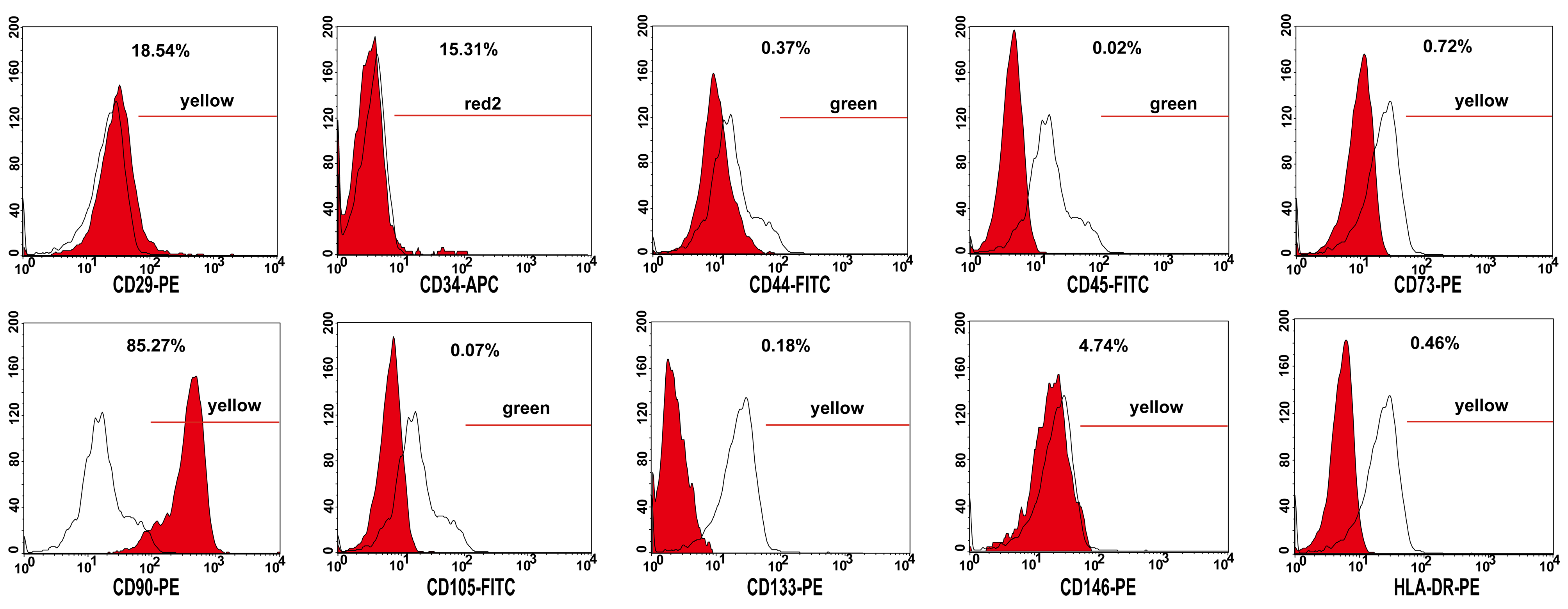

Supplement: Supplementary file 1 — Additional file 1: Figure S1: Flow cytometric analysis of mesenchymal markers of induced pluripotent stem cells (iPSCs). Flow cytometric analysis revealed that iPSCs were positive for CD90, weakly positive for CD29 and CD34, and negative for CD44, CD45, CD73, CD105, CD133, CD146, and HLA-DR. Black histograms represent the isotype controls, and the red solid peak represents the indicated marker. (DOCX 617 KB) [file 13287_2014_465_MOESM1_ESM.docx]
